# Triple interactions for induced systemic resistance in plants

**DOI:** 10.3389/fpls.2024.1464710

**Published:** 2024-11-22

**Authors:** Jihye Jung, Seongho Ahn, Do-Hyun Kim, Myoungjoo Riu

**Affiliations:** Division of Agricultural Microbiology, National Institute of Agricultural Science, Rural Development Administration, Wanju, Republic of Korea

**Keywords:** induced systemic resistance, plant defense mechanism, insect, pathogen, root exudates

## Abstract

Induced systemic resistance (ISR) is a crucial concept in modern agriculture, explaining plant defense mechanisms primed by rhizosphere stimuli and activated by subsequent infections. Biological factors contributing to ISR generally include plant growth-promoting microbes3 (PGPM). *Bacillus* spp., *Pseudomonas* spp., and *Trichoderma* spp. have been extensively studied for their plant growth-promoting characteristics and ISR effect against above-ground pathogens and insect infestations. These phenomena elucidate the bottom-up effects of how beneficial rhizosphere microbes help plants resist above-ground attacks. Conversely, soil microbiome analysis in the rhizosphere of plants infected by above-ground pathogens has shown increased beneficial microbes in the soil, a phenomenon termed 'soil legacy effects'. This represents the top-down effects of above-ground attackers on plants' rhizosphere environments. Interestingly, recent studies have shown that above-ground stimuli not only recruit PGPM in the rhizosphere but also that these PGPM influence plant defense responses against subsequent pathogen infections. This can be seen as a four-step plant defense mechanism involving above-ground attackers, host plants, rhizosphere microbes, and subsequent attacks. This represents an active defense mechanism that overcomes the limitations of sessile plants. This review summarizes plant ISR mechanisms in terms of triple inter-organism interactions and provides molecular evidence for each step.

## Introduction

Similar to the T memory cells in animal immune systems that enhance defense resistance against secondary infections, plants possess a comparable immune strategy known as 'defense priming’. Defense priming is a plant-specific immune strategy for faster and stronger responses to secondary attacks (Hönig et al., 2023). To initiate plant defense priming, primary stimuli such as biological (pathogenic and non-pathogenic microbes), chemical or physical stimuli are required (Jung et al., 2018, 2020). Once the plant's defense mechanism is primed, it develops systemic resistance, which is divided into systemic acquired resistance (SAR) and induced systemic resistance (ISR). SAR and ISR are generally known to have defense responses dependent on the salicylic acid (SA) pathway and the jasmonic acid (JA)/ethylene (ET) pathways, respectively (Choudhary et al., 2007). However, current studies demonstrate that both SA and JA/ET signaling are involved in inducing ISR (Yu et al., 2022).

ISR is primarily primed by non-pathogenic microbes in the rhizosphere, which are regarded as plant growth-promoting microbes (PGPM) (Kloepper, 1978; Hyakumachi, 1994). Numerous studies have shown that PGPM such as *Bacillus* spp. and *Pseudomonas* spp. promote plant growth and trigger ISR, focusing on bottom-up effects and elucidating the influence of below-ground microbes on above-ground plant immune enhancement (Valenzuela-Soto et al., 2010; Beneduzi et al., 2012; Xie et al., 2018).

Furthermore, ongoing microbiome analyses over recent decades have improved our understanding of the interactions between plants and PGPM. Interestingly, above-ground stimuli in plants result in microbiome reshaping in their rhizosphere, recruiting beneficial microbes into their root environment (Lee et al., 2012; Kong et al., 2016; Wang et al., 2021). For example, above-ground whitefly infestation in pepper has been shown to increase colonization of the rhizosphere with beneficial microbes such as *Pseudomonas* spp. Indeed, *Pseudomonas* spp. isolated from pepper roots after above-ground whitefly-infestation showed higher insect-killing activity in *Galleria mellonella* during an *in vitro* mortality assay (Kong et al., 2016). Additionally, aphid infestation in potato (*Solanum tuberosum* L.) leaves leads to lower hatching rates of the endoparasitic nematode *Globodera pallida* in the soil (Hoysted et al., 2018). These phenomena could be regarded as top-down effects, highlighting the influence from the above-ground to the below-ground environment.

Considering both top-down and bottom-up effects, plants actively recruit beneficial microbes into their rhizosphere and utilize them to acquire ISR and resist further pathogen attacks (Wang et al., 2021; Zhu et al., 2021). This can be considered a sessile plant's active defense strategy employing other organisms for their uses. Therefore, in this review, we discuss how plants recruit beneficial microbes in their rhizosphere and use them for defense resistance development, considering both top-down and bottom-up effects.

## Top-down effect: above-ground stimulation alters below-ground microbes

Plants are exposed to both abiotic and biotic stimuli, occurring both above and below ground. How do plant roots perceive signals from above-ground external stimuli, and how do these signals influence below-ground interactions? Recent studies have clearly demonstrated that top-down effects from above-ground bacterial infection or pest infestation can alter the below-ground microbiome (Kong et al., 2016; Wang et al., 2021).

### Above-ground signal perception and long-distance signal transduction

Plants have conserved immune signaling initiated by the recognition of microbe-associated molecular patterns (MAMPs) from external microbes (Muthamilarasan and Prasad, 2013).

Pattern recognition receptors (PRRs) localized on the plant cell surface can recognize MAMPs of pathogenic or non-pathogenic microbes, triggering MAMP-triggered immunity (MTI) in plants (Boller and Felix, 2009; Muthamilarasan and Prasad, 2013). Once PRRs recognize MAMPs, they activate a mitogen-activated protein kinase (MAPK) cascade, which eventually activates WRKY family transcription factors (Pandey and Somssich, 2009; Muthamilarasan and Prasad, 2013). WRKY transcription factors induce the biosynthesis of defense-related phytohormones, such as salicylic acid (SA), jasmonic acid (JA), and ethylene (ET), and promote the production and secretion of antimicrobial compounds into vacuoles (Muthamilarasan and Prasad, 2013).

**Figure 1:**
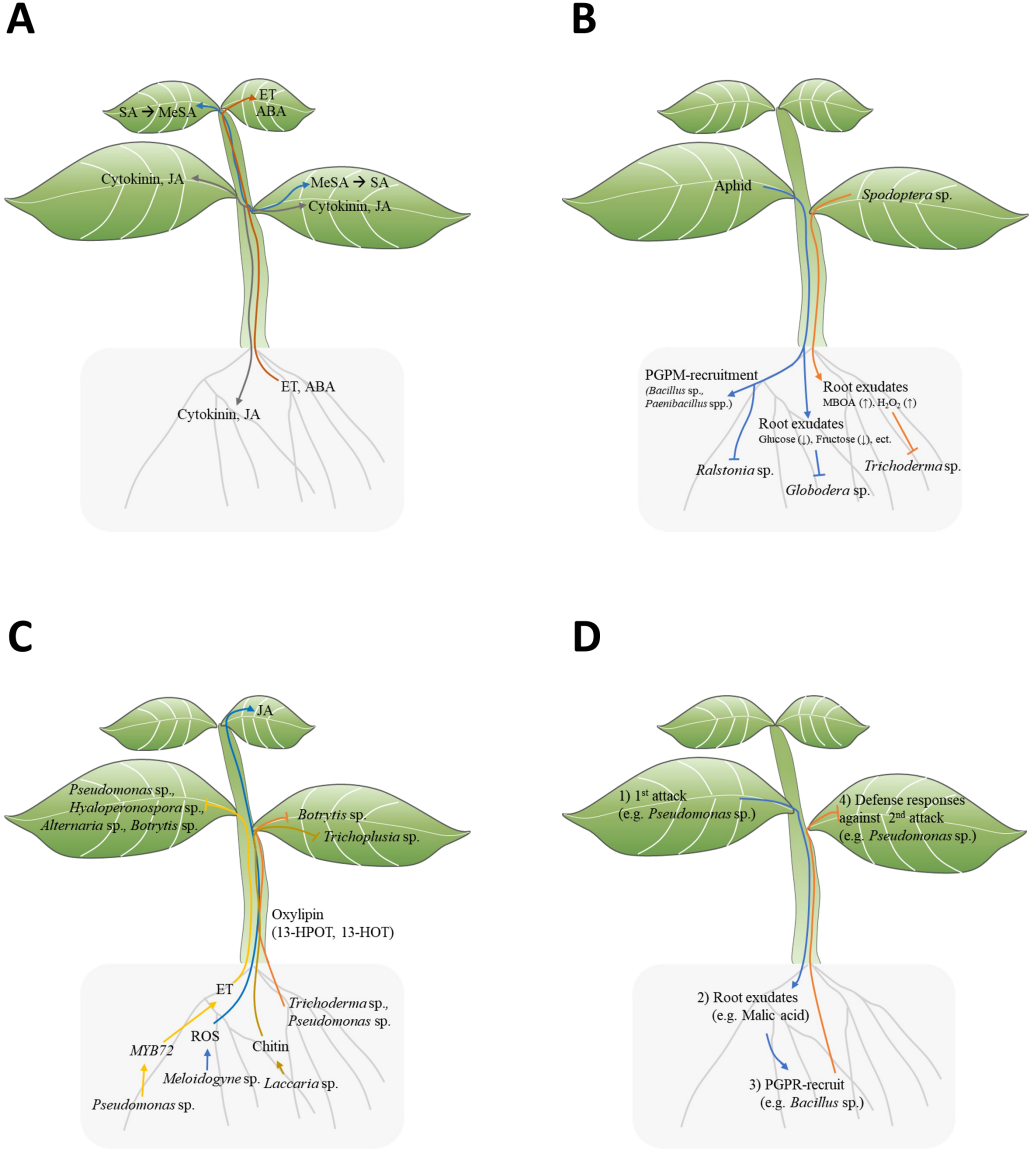
Signal transduction between plant above-ground and below-ground. **(A)** Mobile signal transduction of plant hormones: SA, MeJA, JA, ET, ABA, Cytokinin. (SA, salicylic acid; MeJA, methyl jasmonate; JA, jasmonic acid; ET, ethylene; ABA, abscisic acid). **(B)** Top-down effect of insect infestation on leaves affecting the below-ground environment. **(C)** Bottom-up effect of PGPM on above-ground plant defense responses. **(D)** Triple interactions among above-ground herbivores/pathogens (1st attack), host plants, beneficial rhizosphere microbes, and herbivores/pathogens (2nd attack) in the context of ISR.

Plant hormones like SA, JA, ABA, ET, and cytokinin play roles in plant defense resistance. Notably, JA and cytokinin are reported to be mobile among leaves, from shoot to root, and root to shoot (**Figure 1A**) (Soler et al., 2013). Although SA does not move among leaves, Methyl salicylate (MeSA) moves from cell to cell among leaves or through phloem and is converted to SA in distal leaves (Park et al., 2007) (**Figure 1A**). Among other hormones, ABA is mobile among leaves and from root to shoot, while ET has been reported to move from root to shoot (Soler et al., 2013) (**Figure 1A**). As ET is gaseous hormone, it can diffuse throughout the plant. However, this diffusion refer to general movement rather than directional transport with specific orientation (Binder, 2020). These hormones could potentially induce local defense responses again upon reaching a new location.

In addition to hormones, several long-distance mobile signals have been identified as key players in SAR. These include glycolipids, which are lipid-based molecules acting as signaling compounds in plant defense responses (Chaturvedi et al., 2008). Another important signal is azelaic acid, a dicarboxylic acid that primes plants for enhanced defense activation and contributes to SAR signal generation (Jung et al., 2009). Glycerol-3-phosphate (G3P) also plays a crucial role in SAR as a metabolite synthesized in the cytosol and chloroplasts (Chanda et al., 2011; Shine et al., 2019). Pipecolic acid, a non-protein amino acid, acts as a critical regulator of plant systemic immunity (Návarová et al., 2012). It accumulates in both local and distal tissues following pathogen infection and is essential for SAR activation and priming of plant defenses (Návarová et al., 2012). These diverse molecules work in concert to establish and maintain SAR, enhancing the plant's ability to defend against subsequent pathogen attacks.

The above examples explain mobile phytohormones and small molecules that transfer and function in distal locations. Signals that migrate to the roots could potentially alter the composition of root exudates. These studies support that plant root exudates function to reshape the rhizosphere microbiome, thereby directly affecting belowground evaders or enabling the utilization of surrounding beneficial substances.

### Above-ground stimuli alter root exudation and reshape the rhizosphere microbiome

Above-ground stimuli in plants change below-ground root exudation components, which directly and indirectly lead to the reshaping of the rhizosphere, providing feedback to the plants (Lee et al., 2012; Kim et al., 2016; Hoysted et al., 2018; Friman et al., 2021). Plants with above-ground stimulation may selectively attract specific microorganisms into their rhizosphere. Interestingly, phloem-feeding aphid infestation in pepper (*Capsicum annuum*) leaves attracts beneficial bacteria, such as *Bacillus subtilis* and *Paenibacillus* spp., but not pathogenic bacteria like *Ralstonia solanacearum*, to their roots (**Figure 1B**; **Table 1**) (Lee et al., 2012; Kim et al., 2016). These results suggest that plants actively and selectively reshape the below-ground microbiome in response to above-ground stimuli. How do plants selectively recruit beneficial microbes to the rhizosphere? For example, *Pst* DC3000 infection in *Arabidopsis* leaves led to the secretion of malic acid from their roots (**Table 1**) (Rudrappa et al., 2008). Interestingly, *B. subtilis* strain FB17 exhibits malic acid chemotaxis, suggesting that malic acid may be a key factor in recruiting *B. subtilis* strain FB17 (Rudrappa et al., 2008). Finally, the *B. subtilis* strain FB17 induced systemic resistance against *Pst* DC3000, demonstrating how sessile plants actively trigger defense responses using surrounding beneficial bacteria (**Figure 1D**) (Rudrappa et al., 2008).

**Table 1:** Summary of Results from top-down and bottom-up effects.

Host	Inducer	Result in below-ground/above ground	References
Pepper	Aphid	-Attracts beneficial bacteria, such as *B. subtilis* and *Paenibacillus* spp., but not pathogenic bacteria *R. solanacearum*	(Lee et al., 2012; Kim et al., 2016)
*Arabidopsis*	*Pseudomonas syringae* pv. tomato	-Higher levels of amino acids, nucleotides, and LCOAs (C > 6) - lower levels of sugars, alcohols, and SCOAs (C ≤ 6)	(Yuan et al., 2018)
Potato	Aphid	-Reduced glucose and fructose contents	(Hoysted et al., 2018).
Maize	*Spodoptera frugiperda*	-Increased NSCs, MBOA, and the reactive oxygen species H_2_O_2_ -Growth inhibition of the rhizosphere fungus *T. atroviride*	(Adame-Garnica et al., 2023)
*Arabidopsis*	*Pst* DC3000	-Secretion of malic acid -Recruiting *B. subtilis* strain FB17	(Rudrappa et al., 2008).
*Arabidopsis*	*myc2* mutant	-Increased abundance of *Streptomyces*, *Bacillus*, and *Lysinibacillus* taxa in the rhizosphere	(Carvalhais et al., 2015)
Tomato	*rin mutant*	*-*Increased *RIN* transcription factor, 3-hydroxyflavone and riboflavin, Actinobacteria (*Streptomyces*) in the rhizosphere	(Yang et al., 2023)
Tomato (resistant cultivar)	*Fusarium oxysporum* or fusaric acid (FA)	-Altered the root exudate components of tomato between susceptible and resistant cultivars -Enhancing colonization of disease-suppressive bacteria, *Sphingomonas* sp., in the resistant cultivar	(Jin et al., 2024)
*Arabidopsis*	*P. capeferrum* WCS358	MTI supersession: flg22-mediated *Arabidopsis* root immunity by producing gluconic acid	(Yu et al., 2019a)
*Arabidopsis*	*Pseudomonas* sp. WCS365	MTI supersession: *MorA* and *SpuC* dependent biofilm inhibition	(Liu et al., 2018)
*Arabidopsis*	*P. fluorescence* WCS417r	-MYB72-Ethylene dependent ISR against *P. syringae* pv tomato, *H. parasitica*, *A. brassicicola*, and *B. cinerea*	(Van der Ent et al., 2008
*Arabidopsis*	*L. bicolor*	-*L.bicolor (*or chitin*)* triggers ISR in *Arabidopsis* against *T. ni*	(Vishwanathan et al., 2020)
Rice	*Chitin*	-Cytokinin signaling downregulation -> cell wall component alteration -> ISR against *B. oryzae*	(Takagi et al., 2022)
*Arabidopsis*	*Pseudomonas* spp. *fluorescent*	-*SYP123* dependent ISR marker genes (*PR1*, *MYC2*, and *PDF1.2*) expression	(Rodriguez-Furlán et al., 2016; Zhu et al., 2022)
Tomato	*Meloidogyne incognita*	*-RBOH1*, *GLR3.5*, and *MPK1/2*-dependent ROS signal transduction (root to leaves) -> JA accumulation in leaves -> resistance to *M. incognita*	(Wang et al., 2019)
Maize	*Trichoderma virens* trigger	-12-OPDA and KODA biosynthesis leading to ISR	(Wang et al., 2019)
Tomato	*Pseudomonas putida* BTP1	-ISR against *B. cinerea* by accumulating two antifungal oxylipin, free 13-hydroperoxy-octadecatrienoic and 13-hydroxy-octadecatrienoic acids	(Mariutto et al., 2011)
Maize	*Bacillus velezensis* FZB42	-ISR by regulating nuclear factor Y transcription factor regulated by miR169 family	(Xie et al., 2019)

Above-ground attacks on plant leaves may influence plant root exudate. Recent studies have shown that root exudate components change following above-ground infections (Doornbos et al., 2012; Kim et al., 2016). Root exudation profiles in *Pseudomonas syringae* pv. tomato (*Pst*)-infected *Arabidopsis* exhibited significantly higher levels of amino acids, nucleotides, and long-chain organic acids (LCOAs) (C > 6) and lower levels of sugars, alcohols, and short-chain organic acids (SCOAs) (C ≤ 6) (**Table 1**) (Yuan et al., 2018). Interestingly, since exogenous amino acids and LCOA application to plants showed disease-suppressive responses, the *pst*-induced root exudation may function as plant’s defense enhancement (Yuan et al., 2018). Similarly, aphid-infested potato leaves also showed reduced sugar contents in root exudates (Hoysted et al., 2018). Root exudates from aphid-infested potato (*Solanum tuberosum* L.) showed reduced glucose and fructose contents, resulting in lower hatching rates of below-ground endoparasitic nematode *Globodera pallida* (**Table 1**; **Figure 1B**) (Hoysted et al., 2018). However, sugar supplementation did not recover the hatching potential of *G. pallida*, indicating that root exudation may involve other important factors for egg hatching (**Figure 1B**) (Hoysted et al., 2018). In the case of chewing insect stimuli, leaf area and biomass decrease by *Spodoptera frugiperda*-infested maize leaves correlated with changes in root exudate compounds (Adame-Garnica et al., 2023). The degree of *S. frugiperda* infestation modulated the root exudation contents, non-structural carbohydrates (NSCs), 6-methoxy-2-benzoxaxolin-2-one (MBOA), and the reactive oxygen species H_2_O_2_, leading to the growth inhibition of the rhizosphere fungus *Trichoderma atroviride* (**Table 1**; **Figure 1B**) (Adame-Garnica et al., 2023). This result clearly demonstrates the top-down effect of above-ground insect attacks, showing how they influence root exudation and, consequently, effect on rhizosphere fungus.

### Molecular evidence of root exudate-mediated rhizosphere microbiota shaping and disease resistance in plants

Recent studies have provided molecular evidence of changes in root exudates. For example, JA is a critical hormone involved in plant defense responses, has been shown to influence root exudation. Root exudates from *Arabidopsis* JA mutants, such as *myc2* and *med25*, showed reduced levels of asparagine, ornithine, and tryptophan (Carvalhais et al., 2015). Additionally, these *myc2* mutants had an increased abundance of *Streptomyces*, *Bacillus*, and *Lysinibacillus* taxa in the rhizosphere, suggesting that JA-dependent root exudates alterations affect microbiome communities (Carvalhais et al., 2015).

A recent study indicated that the *ripening-inhibitor* (*RIN*) transcription factor plays a role in assembling disease-suppressive rhizosphere microbiota by altering root exudates. *RIN* tomato mutant exhibited lower concentration of 3-hydroxyflavone and riboflavin in their root exudates, leading to reduction in pathogen-suppressing Actinobacteria (*Streptomyces*) in the rhizosphere (Yang et al., 2023). The disease suppressiveness of the *rin* plant microbiome was restored by complementing with 3-hydroxyflavone and riboflavin (Yang et al., 2023).

Interestingly, plant root exudates also changed in response to fusaric acid (FA), produced by *Fusarium oxysporum* (Jin et al., 2024). FA differently altered the root exudate components of two tomato cultivars; *F. oxysporum* f. sp. *lycopersici* resistant Z19 and susceptible D72. FA enhanced colonization of disease-suppressive bacteria, *Sphingomonas* sp., in the resistant cultivar Z19 (Jin et al., 2024). These microbiome changes provided feedback, leading to defense resistance to *F. oxysporum* in the resistant cultivar Z19 (Jin et al., 2024).

## Bottom-up effect: signal recognition and transduction from root to leaf

For a mutually beneficial relationship between plants and microbes, plants need to recognize certain microbes as symbiotic friends rather than enemies to fight. However, MTI is induced regardless of whether microbes are beneficial or harmful. Beneficial microbes, therefore, need to suppress or evade plant immune responses to establish a symbiotic relationship (Yu et al., 2019b). How do beneficial microbes colonize the rhizosphere while suppressing the plant immune system? Some evidence suggests that beneficial microbes suppress plant immunity in roots, facilitating their colonization in the rhizosphere.

### Symbiotic relationship by MTI suppression

Flg22 is highly conserved domain of bacterial flagellin, recognized as a MAMP by the plant FLS2 receptor, resulting in MTI (Felix et al., 1999). *Pseudomonas capeferrum* WCS358, a well-known PGPM (Meziane et al., 2005), suppresses flg22-mediated *Arabidopsis* root immunity by producing gluconic acid and shows enhanced colonization in *Arabidopsis* roots compared to gluconic acid production mutants (*pqqF*::Tn5 and *cyoB*::Tn5) (**Table 1**) (Yu et al., 2019a). Another study, using a high-throughput transposon (Tn-Seq) screening system in *Pseudomonas* sp. WCS365, found that *morA* and *spuC* mutants induced MTI, enhanced biofilms formation, and inhibited the growth of *Arabidopsis* compared to the wild type (**Table 1**) (Liu et al., 2018). *MorA* (encoding phosphodiesterase) and *SpuC* (encoding putrescine aminotransferase) are related to the suppression of biofilm formation. This biofilm inhibition may be one of the microbes' strategies for evading MTI and fitting within the rhizosphere.

Considering that ISR occurs through the recognition of microbial-derived factors by the plant, the symbiosis between plants and microbes is crucial (Zamioudis and Pieterse, 2012). However, current studies suggest that some ISR responses might not require a symbiotic association (Vishwanathan et al., 2020). For example, the ectomycorrhizal fungus *Laccaria bicolor* can trigger ISR against the insect *Trichoplusia ni* in *Arabidopsis*, a nonmycorrhizal plant (Vishwanathan et al., 2020). Therefore, the crucial factor may lie more in how effectively the plant responds to ISR elicitors secreted by microbes, rather than the duration of the plant-microbes symbiotic relationship.

### Microbe elicitor-plant recognition and signal transduction

Plants may recognize signals from rhizosphere microbes for inducing ISR. Then, what happens in plant roots for initiating ISR signaling?

*Pseudomonas fluorescens* WCS417r is known for inducing ISR against a broad spectrum of pathogens in *Arabidopsis*. However, *P. fluorescence* WCS417r showed impaired ISR in *myb72-1* and *myb72-2 Arabidopsis* mutant against *Pseudomonas syringae* pv tomato, *Hyaloperonospora parasitica*, *Alternaria brassicicola*, and *Botrytis cinerea* (**Table 1**; **Figure 1C**) (Van der Ent et al., 2008). Indeed, the root-specific transcription factor MYB72 appears to be a convergence node, as it is essential for early signaling in ISR induction by *P. fluorescence* WCS417r and *Trichoderma asperellum* T34 (Van der Ent et al., 2008; Segarra et al., 2009). The author suggest MYB72 is upstream of ET signaling, as WCS417r activated MYB72 in ethylene-insensitive *ein2-1* plants, and exogeneous ethylene precursor 1-aminocyclopropane-1-carboxylate (ACC) induced ISR responses in *myb72-1* mutants (**Figure 1C**) (Van der Ent et al., 2008).

Chitin, one of the MAMPs of microbes, has been shown to induce ISR (Vishwanathan et al., 2020; Takagi et al., 2022). *L.bicolor* triggers ISR in *Arabidopsis* against *Trichoplusia ni* (**Figure 1C**) (Vishwanathan et al., 2020). Molecular evidence suggest that chitin derived from *L.bicolor* is an ISR-inducing molecules, as heat-killed *L. bicolor* or chitin also trigger ISR, but the chitin receptor mutant *cerk1-2* could not trigger ISR by *L.bicolor* or chitin (**Table 1**) (Vishwanathan et al., 2020). Another study showed that chitin-induced ISR against *Bipolaris oryzae* in rice is related to down-regulated cytokinin signaling, resulting in alterations of cell-wall components (**Table 1**) (Takagi et al., 2022). Therefore, chitin-induced ISR is mediated by perturbation in cell-wall biogenesis in leaves.

Regarding PGPM interactions with plant roots, the root hair is the first part of the plant to interact with microbes. Root hair-specific syntaxin gene *SYP123* is suggested to be necessary for PGPM-triggered ISR, as ISR marker genes (*PR1*, *MYC2*, and *PDF1.2*) did not increase in *syp123 Arabidopsis* mutants in response to beneficial *Pseudomonas* species (Rodriguez-Furlán et al., 2016; Zhu et al., 2022).

In the case of root-to-shoot communications, the root-knot nematode (RNK) *Meloidogyne incognita* induces the transition of ROS signals from root to leaves of tomato, resulting in JA accumulation in leaves (**Figure 1C**) (Wang et al., 2019). The *M. incognita*-induced signal transduction involves *RBOH1*, *GLR3.5*, and *MPK1/2*-dependent JA accumulation, as JA accumulation in leaves was abolished in grafting experiments with scion of the mutant *GLUTAMATE RECEPTOR-LIKE 3.5* (*GLR3.5*), *RESPIRATORY BURST OXIDASE HOMOLOG1* (*RBOH1*), and plant silenced for *mitogen-activated kinases1* (*MPK1*) or *MPK2* (Wang et al., 2019). The RNK *M. incognita* induced-JA accumulation in leaves transfers to the roots, triggering resistance to RNK (**Table 1**) (Wang et al., 2019).

Oxylipins are oxidized lipid signals that regulate plant physiological responses to abiotic and biotic stress, including defense responses against pathogens and insects (Wang et al., 2019). Several non-jasmonate oxylipins have been identified as crucial regulators for ISR in various plant species. In maize, two xylem-mobile oxylipins, 12-oxo-phytodienoic acid (12-OPDA) and an γ-ketol of octadecadienoic acid (KODA), play important roles in ISR (Wang et al., 2019; Carella, 2020). Additionally, two γ-ketols, 12-Oxo-9-hydroxy-10(*E*)-octadecenoic acid (9,12-KOMA) and 12-Oxo-9-hydroxy-10(*E*),15(*Z*)-octadecadienoic acid (9,12-KODA), have been identified as ISR priming agents in maize (Wang et al., 2020). The small secreted protein Sm1 from PGPM *virens* triggers ISR regulating 12-OPDA and KODA biosynthesis in maize plants (**Table 1**) (Wang et al., 2019). Similarly, *Pseudomonas putida* BTP1 induced ISR against *Botrytis cinerea* by accumulating two antifungal oxylipin, free 13-hydroperoxy-octadecatrienoic (13-HPOT) and 13-hydroxy-octadecatrienoic acids (13-HOT), in tomato (**Figure 1C**; **Table 1**) (Mariutto et al., 2011).

Recently, microRNAs have also been suggested as mobile signals for plant-microbe interactions (Xie et al., 2019). *Bacillus velezensis* FZB42, which is reported to induce ISR, showed that four miRNAs, zma-miR169a-5p, zma-miR169c-5p, zma-miR169i-5p, and zma-miR395b-5p, belonging to the miR169 family, are associated with triggering ISR by regulating nuclear factor Y transcription factor in maize (**Table 1**) (Xie et al., 2019).

## Perspectives

This study explored the triple interaction among above-ground herbivores/pathogen (1^st^ attack), host plants, beneficial rhizosphere microbes PGPM, and herbivores/pathogen (2^nd^ attack) in the context of ISR. ISR is triggered by beneficial microbes interacting with plant roots, enhancing the plant's defenses against pathogens and pests. In this review, we focused on how PGPM enhance systemic resistance in plants through ISR and activate defense mechanisms against herbivores and pathogens. Many previous studies have shown that plants can actively assemble beneficial microbes in the rhizosphere according to their needs. Even in the absence of a symbiotic associations, these microbes can induce ISR in plants.

There are naturally occurring complex interactions: (1) plant leaves are exposed to pathogens and insects (above ground microbe’s first attack-plant interaction), and (2) plant roots interact with various microbes in their rhizosphere (below ground microbes-plant interaction). We propose a concept of 'triple interactions' by adding the plant's active immune response, specifically ISR, for a second above-ground attack. This raises the question: How can we apply these complex plant immune systems to agriculture?

Since plants can actively gather the microbes they need in the rhizosphere, it is possible to study the types of beneficial microbes that can control pests and pathogens that are currently difficult to manage. For instance, by inoculating plants with target pests or pathogens and then observing the changes in the microbial community over time, it may be possible to identify microbes that the plant recruits to combat the target pest/pathogen. Alternatively, since root exudates released under specific stress conditions ultimately gather beneficial microbes, identifying the root exudate components that attract PGPM would also be beneficial for future agricultural applications.

Unlike animals, plants do not have T-cells and cannot be vaccinated in the traditional sense. However, by actively recruiting beneficial microbes to the rhizosphere after an attack, plants can enhance their systemic resistance, functioning similarly to a vaccine. Of course, there are also cases where microbial communities harmful to the plant assemble in response to an above-ground attack. Therefore, future studies could investigate whether there is a threshold beyond which a stressed plant's ability to recruit beneficial microbes becomes ineffective. If this threshold is exceeded, it could potentially neutralize the plant's capacity to assemble beneficial microbial communities. Moreover, it is important to acknowledge that not all plants can acquire immunity against every pest or pathogen they encounter. This limitation means that the concept under discussion may not apply universally. Further research is needed to understand the boundaries of a plant's ability to recruit beneficial microbes under various stress conditions and how these limitations impact overall plant health and resistance.
